# Are the Head and Tail Domains of Intermediate Filaments Really Unstructured Regions?

**DOI:** 10.3390/genes15050633

**Published:** 2024-05-16

**Authors:** Konstantinos Tsilafakis, Manolis Mavroidis

**Affiliations:** 1 Center of Basic Research, Biomedical Research Foundation, Academy of Athens, 11527 Athens, Greece; emavroeid@bioacademy.gr; 2 Department of Biochemistry and Biotechnology, University of Thessaly, Biopolis, 41500 Larissa, Greece

**Keywords:** intermediate filaments, low-complexity regions, structure

## Abstract

Intermediate filaments (IFs) are integral components of the cytoskeleton which provide cells with tissue-specific mechanical properties and are involved in a plethora of cellular processes. Unfortunately, due to their intricate architecture, the 3D structure of the complete molecule of IFs has remained unresolved. Even though most of the rod domain structure has been revealed by means of crystallographic analyses, the flanked head and tail domains are still mostly unknown. Only recently have studies shed light on head or tail domains of IFs, revealing certainsecondary structures and conformational changes during IF assembly. Thus, a deeper understanding of their structure could provide insights into their function.

## 1. Introduction

**Figure 1:**
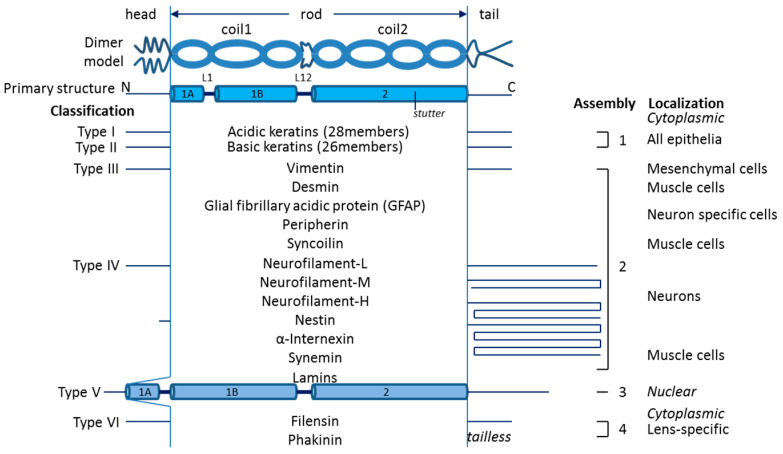
Intermediate filament proteins have been classified into six distinct types based on sequence identity and tissue distribution [10,18,19]. Assembly groups are separated by different kinds of polymerization. All intermediate filament proteins have a characteristic tripartite structure, consisting of a highly α-helical central rod domain that is flanked by non-α-helical head and tail domains. The linker L12 acts as a flexible hinge. The rod domain consists of the heptad repeats that are the signature of α-helical proteins. The structure and length of the central rod domain are highly conserved in vertebrates, except from the nuclear lamins, which contain six extra heptads in the 1B segment, and thus coil1B is larger in lamins than the other IFs. A four-residue insertion in segment 2B produces a discontinuous heptad-repeat pattern (“stutter”) which is highly conserved in all IF proteins. The variability of intermediate-filament proteins lies in the length and sequence of the head and tail domains (for example, the short head and long tail domains of nestin [20] or the tailless phakinin [21] or keratin19 [22]), that are thought to be involved in regulating the interactions between intermediate filaments and other proteins.

Throughout evolution, living cells have developed the cytoskeleton, a mechanical network providing both stability and dynamics. Cytoskeletal filaments are represented by microtubules (MTs), intermediate filaments (IFs), and microfilaments (MFs). In metazoans, ΙF proteins are encoded by large gene families and even though they exhibit different primary structure and biochemical properties, they all share a common structural framework while maintaining their tissue specificity [1]. About 70 genes have been found and have been sequenced in humans which translate to different types of IF proteins (Figure 1), as well as their orthologs in various species [2,3,4]. Cytoplasmic IF genes were first identified in vertebrate genomes, while the nuclear IFs, which are called lamins, are universally present in metazoans. Lately, IF-like genes have also been identified in invertebrate genomes [5,6]. It is thought that the archetypical cytoplasmic IF protein arose in eukaryotic evolution from a mutated lamin gene which lost two signal sequences related to lamin functionality (the nuclear localization signal and the CaaX box) [7]. Interestingly, lamin evolution has important implications with regard to the evolution of the nuclear envelope [8]. The remarkable adaptability of the IF structure makes these proteins powerful building blocks involved in the development of new cellular shapes and functions.

IF proteins exhibit a common tripartite structure which consists of a central α-helical rod domain, surrounded by the variable non-helical and low-complexity head and tail domains (Figure 1). The characteristic central α-helical rod domain is a signature of all IF proteins and contains the subregions and linkers coil1A, L1, coil1B, L12, and coil2 in line. The basic unit during the assembly of IFs seems to be the dimer, as single molecules tend to dimerize and create an α-helical coiled coil (CC) by their rod domains [9,10]. IFs have unique properties such as a lack of polarity, high flexibility, and extensibility, in contrast to the much more stiff and fragile MTs and MFs [11,12,13]. However, this specific structure of intermediate filaments makes their isolation and crystallization challenging, explaining the limited structural data. Thus, the crystallization of a full IF monomer or dimer has not been achieved yet. Nevertheless, the conserved rod domain is described in various intermediate filaments by means of (X-ray) crystallography [14,15,16]. It has been shown that the coil2 region of vimentin, a typical type III cytoplasmic IF protein, is continuously helical without the interconnection of linker L2 [17], as was previously thought. Moreover, linker L1 was found to be helical too, but without being involved in the coiled-coil formation [14]. Likewise, unlike previous predictions, the linker L12 in lamins also seems to be helical [16].

In the present study, we describe the known and current findings on structural data of the less studied head and tail domains of IF proteins, the significance of those domains in assembly process, and eventually their role in IF function.

### 1.1. The Role of Head and Tail Domains in IF Assembly

The main role of the head domain of IF proteins seems to be structural, as it is mainly important for the correct composition and organization of the final form of the intermediate filaments. While the N-terminal head domains seem to control IF assembly and stabilization, the tails are thought to regulate the lateral packing and stabilization of higher order filament structure [1]. Assembly varies among different types of intermediate filament proteins; type I acidic keratins and type II basic keratins assemble only in heterodimers, while type III and IV IFs assemble both in homodimers and heterodimers (Figure 1). On the other hand, type V lamins start the assembly process with head-to-tail interactions and they do not co-polymerize with other types of IF proteins. Lastly, type VI IFs assemble in hetero-polymer IFs to uniquely form the lens-specific “beaded-chain filaments” which consist of filensin/phakinin hetero-oligomers [23]. In general terms, the cytoplasmic IFs initially assemble in parallel dimers and then in anti-parallel tetramers. Next, the tetramers interact laterally to form protofilaments (or unit-length filaments, ULFs) which anneal longitudinally, before the final maturation phase that involves radial compaction—as originally proposed by Herrmann and Aebi in 1998 [3]. However, the keratin IF formation progress is different. They assemble very rapidly into polymers, and their lateral association and longitudinal annealing occur almost simultaneously [24].

**Figure 2:**
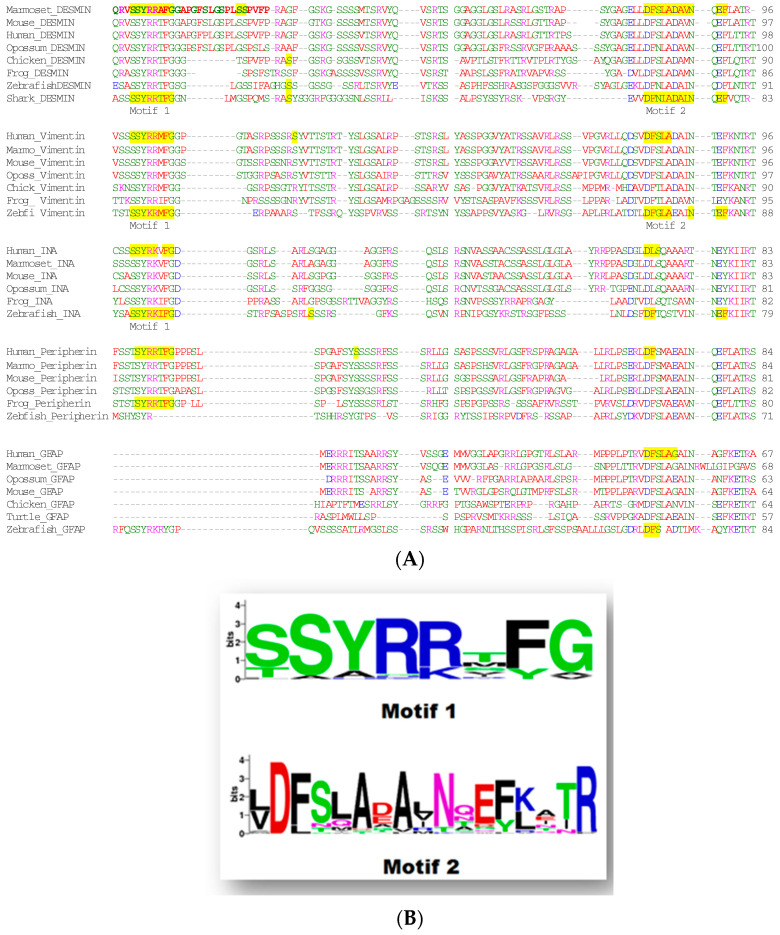
(**A**). Multiple sequence alignment of the head domain of type III intermediate filament proteins (desmin, vimentin, peripherin, and glial fibrillary acidic protein) and type IV α-internexin from different species. (**B**). The alignment indicates two highly conserved regions, motif-1 and motif-2, in the head domain at the beginning and at the end, respectively, with the exception of GFAP which lacks motif-1 but preserves another motif with similar characteristics [35]. The alignment of sequences was performed with MUSCLE (multiple sequence comparison by log-expectation) [36] and visualized with Jalview [37]. The optimization of the alignment was carried out with the computational program MacClade [38]. Colors correspond to Jalview criteria.

The existence of the head domain is important even from the first steps of assembly and especially at the stage of tetramers in type III IFs [25,26]. Notably, an in vitro study revealed that in the dimer formation of vimentin, the head domain folds back and interacts with coil1A [27]. This interaction is very significant since when it is hindered, the formation of intermediate filaments is disrupted [28] or aggregates are observed [29]. Interestingly, the interacting amino acid G17 from vimentin’s head domain [27] resides in the extremely preserved nonapeptide motif “SSYRRTFGG” (Figure 2), which has been shown to be necessary for the assembly of IFs [30,31]. Another motif, albeit less conserved, is located at the end of the head domain of type III intermediate filaments and type IV α-internexin protein (Figure 2). Recent data support that during vimentin’s assembly, the head domain interacts in intra-dimeric and inter-dimeric ways to form tetramers [32]. Afterwards, at the elongation phase, the ULFs are joined longitudinally through head-to-tail interplay [3,33], similarly to lamin filament formation where the head-to-tail interaction happens first [34].

More specifically, during the formation of the premature ULFs of the well-studied vimentin, octamers seem to interact laterally through the tail domains and centrally through interactions of the head domains, where coil 2 dimers shape the outer vimentin IF surface, whereas coil 1A–1B dimers predominantly coat the inner surface of the filaments [39]. In this way, vimentin IFs incorporate the low-complexity amino terminus domains within a highly structured helical structure, thereby creating an additional level of structural complexity and filament assembly that relies on transient molecular interactions [39]. The connection of the flexible terminal domains enhances the construction of a high-strength and -elasticity biopolymer [39,40]. Nevertheless, vimentin filaments that polymerize within the cell appear to exhibit polymorphisms in conformation. Indeed, two configurations of vimentin filaments have been described, with the main difference being the existence or lack of an internal amyloid-like fiber inside the lumen of ULFs which is constituted through the interaction of the head domains. However, the researchers point out that somehow both the head and tail domains of intermediate filaments remain accessible for modifications and interactions [39,40]. The assembly process of intermediate filaments seems to be dynamic and requires numerous interactions in a very coordinative and hierarchical manner, where the low-complexity regions are equally necessary.

On the other hand, type I and II keratin molecules intertwine in parallel, forming heterodimers. The model of keratin K1/K10 dimer is formed in a similar way to vimentin as the head domain folds back and interacts with the rod domain, forming a globular structure [41]. The head domain is also involved in the next stages of keratin filament formation [24]. Among type IV IFs, nestin is incapable of forming homopolymers because of its particularly short head domain, and thus it usually co-assembles with other type III or IV cytoplasmic IFs [42]. Moreover, the α-internexin head domain is significant in self-assembly and co-assembly with neurofilament proteins [43]. The head domains of nuclear lamins are relatively short compared to the head domains of cytoplasmic IF proteins, but they are equally important [44,45]. An in vitro analysis has shown that the last twenty residues of the head domain of lamin A are essential for the construction of the head-to-tail polymers [45]. In addition, deletion mutations in the C-terminal CaaX domain of lamin B1 and deletion mutations in either the head or CaaX domain of lamin A form intranuclear aggregates and disrupt the endogenous lamins A/C without affecting lamins B1/B2 [46]. The head-to-tail association involves an “overlap” of the highly conserved rod domain, and interactions with the head domain are vital for dimer formation, probably through electrostatic interactions [16,47]. Finally, in the exceptional case of type VI IFs, it seems that the tail domain of filensin is necessary for beaded filament formation with the naturally tailless phakinin [48,49], while the partial truncation of the N-terminal domain of filensin does not affect filament formation [49].

### 1.2. Structural Data

A unique feature of vimentin and sequence-relative IF proteins (such as desmin and neurofilaments) is that they preserve a small pre-coil domain (pcd) in the head domain that mainly contains amino acids with a high probability for α-helix formation, but without this structure having been determined. This motif is absent from keratins and lamins [1,50]. Depending on the stage of the IF assembly, the pcd interacts with the rod domain through electrostatic forces, like H-bonds. Through these transient interactions, the head domain may play an essential role in preventing off-pathway interactions and guiding the tetramers to the productive oligomerization pathway [51]. Recent research reinforces the assumption that the head domain must exhibit some kind of structural order, where the isolated amino-terminal head domains form an unstable cross β-strand secondary structure that promotes self-association and facilitates the assembly of desmin and NFL (neurofilament-light), as was shown using solid-state NMR (ss-NMR) spectroscopic studies [52,53]. Mutations in phosphosites of desmin and NFL increase the self-association of the head domains, resulting in the formation of immature polymers, a disrupted network of IFs, and the formation of aggregates [52]. On the other hand, some IFs, particularly keratins and nuclear lamins, have glycine-rich repeating peptides in the head and tail domains that form a “glycine loop” structural motif [54]. Especially in keratins, a subdivision of the head domain has been proposed which includes three subdomains: subdomain E1; a variable glycine-rich subdomain, V1; and a region of sequence homology close to the rod domain, H1. In a similar way, the corresponding subdomains of the tail domain are E2, V2, and H2. One modeling analysis of keratins K1/K10 in octamer assembly proposed an aromatic zipper mechanism that brings together two tetramers forming a tetrameric terminal domain complex through the interaction of glycine loop regions of head and tail domains [55]. Furthermore, it is known that the conserved H1 subdomain of the head domain of type II keratin 1 is indispensable in the early stages of keratin K1/K10 intermediate filament formation [56].

Nevertheless, the only crystal structure of the low-complexity regions in IFs is derived from the lamin tail domain and indicates that the lamin-specific motifs of Ig-like fold motifs interact with each other in dimer formation and create a continuous β-fold sheet structure [57], which indicates some kind of secondary structure during the process of assembly. Another piece of information about the complex interaction of the tail domains in filaments comes from type IV neurofilaments and α-internexin long tails, which have recently been investigated using small-angle X-ray scattering experiments, yielding a concept that the protruding tails conform in a “bottle brush” network [58]. Additionally, a study based on the electron paramagnetic resonance (EPR) analysis of vimentin tail domain revealed some structural order like the formation of a β-hairpin structure and conformational change during filament elongation and final assembly process [59]. Moreover, an in silico analysis of desmin’s head domain has predicted a 3D model which consists of both α helical motifs and β sheets, which contradicts a previous theory that assumed that the head domain is unstructured and of a low level of complexity [60]. In support of these predictions, a previous infrared spectroscopy study showed that a major part of the head domain of desmin consists of β-sheet formations [61].

### 1.3. Interactions

Under the assumption that both the head and tail domains protrude from mature intermediate filaments, they appear to interact with other proteins or organelles. For example, desmin’s head domain interacts with myospryn [62], and thus it is connected to lysosome function as myospryn binds to dysbindin, a component of the biogenesis of lysosome-related organelles complex 1 (BLOC-1), which regulates protein trafficking and organelle biogenesis [63,64]. Recently, it was shown that desmin’s head domain is necessary for interactions with the lysosomal protein saposin D and the mitochondrial protein NDUFS2 (NADH ubiquinone oxidoreductase core subunit S2) [60], a core subunit of complex I, and therefore plays a significant role in mitochondrial function. At the same time, it has been shown that desmin can also bind directly to mitochondria in vitro through the head domain [65]. These observations are in line with a report suggesting that the N-terminal domain of vimentin, another type III IF protein like desmin, is not only responsible for interaction with mitochondria but it also regulates the mitochondrial membrane potential [66].

The cytoskeletal network of intermediate filaments is essential for tissue integrity, and plakin proteins contribute to the formation of cell–cell and cell–matrix junctions and connections to organelles such as mitochondria and nuclei by linking intermediate filaments, actin microfilaments, and microtubules. Initially, it was shown that the head domain of most type II keratins interacts with desmoplakin [67,68], but a recent study indicated that coil1 from IFs I-III is necessary for the interaction [69]. However, it should be emphasized that desmoplakin binds more strongly to full-length IF proteins [69], and as the head domain is important for the proper assembly of intermediate filaments, it could also be considered significant and maybe act synergistically with the desmoplakin binding. Another plakin family protein, plectin, interacts with desmin’s and vimentin’s rod domains [70]. Plectin also binds to keratin K5/K14 rod domains and the polymerization of keratin filaments significantly enhances the binding [71], noting again the importance of the assembly process. Although the associations between IFs and plakin proteins are strong, there are regulatory mechanisms that temporarily modulate those interactions. In fact, posttranslational modifications (PTMs), such as phosphorylation events, control the assembly stage of IFs and thus control the interactions between different proteins affecting IF function in processes such as cell migration, cell mitosis, and stress responses [72]. Furthermore, the head domain acts as a recipient of multiple post-translational modifications regulating the dynamic assembly–disassembly process of the IF network and contributes to maintaining the structural and functional integrity of IFs [72,73,74,75]. Important interactions between IFs and other cellular components and structures are catalyzed through PTMs [76]. In addition, a plethora of mutations in intermediate filament genes, that affect PTMs, some of which are located in the head domain (as is the case with desmin [19]), are related to different diseases [74,76].

Meanwhile, the border region between the head and 1A domain can be a genetic hot spot, carrying pathogenic mutations in different IF-encoding genes like *KRT5*, *GFAP*, or *LMNA*, encoding, respectively, keratin 5, glial fibrillary acidic protein, and lamin A/C [77,78,79]. Interestingly, *DES* mutations are differently associated with desminopathies, depending on the domain they affect, as mutations in the head or tail domain of desmin are mostly identified in patients with isolated cardiological signs, while mutations in the rod 2B domain affect both the skeletal and cardiac muscle of patients, according to a phenotype–genotype correlation meta-analysis [80]. IFs also seem to play a pivotal role in signaling pathways [81], especially through the binding interactions of signaling components to both the head and tail domains of IF proteins [18,74,82]. For example, cdk5 activity is regulated by the scaffolding of cdk5/p35 onto nestin through tail domain interactions [83].

Remarkably, IF proteins and especially type I-IV IF proteins seem to bind to nucleic acids through their N-terminal domains and thus maybe participate in the regulation of genomic events [84]. These studies referred to the cytokeratins K8, K18, and K19 from type I and II; the proteins desmin, glial fibrillary acidic protein (GFAP), peripherin, and vimentin from type III; and the neurofilament protein L (NFL) from type IV. Also, the interactions show some similarities, i.e., all of the head domains were found to possess both a majority of arginine residues and multiple aromatic residues which can be crosslinked to an oligonucleotide [84]. In vivo, there is a pathological condition where the disruption of the vimentin IF network, followed by the activity of the HIV-1 protease, results in the entry of free vimentin head domain peptides into the nucleus and the perturbation of the organization of nuclear chromatin [85]. Regarding type V of IFs, the tail domain of lamin A/C, and especially the Ig-like fold region, interacts and forms a ternary complex with emerin, a protein of the inner nuclear membrane, and with BAF (Barrier-to-Autointegration Factor), a chromatin-associated protein, according to a 3D-structure scope [86]. Mutations in this region of lamin A/C are linked to muscle diseases [87]. On the other hand, an in vitro study shows that the head and coil1 domains of lamin B1 are necessary for the interaction with p53-binding protein (53BP1), which plays a pivotal role in genome repair and stability [88].

### 1.4. Low-Complexity Regions

Intermediate filament proteins have been shown to have low-complexity regions (LCRs) in their head and tail domains. This is not a unique feature, as over 20% of eukaryotic proteomes include polypeptide sequences that are of low complexity [89]. The majority of proteins carrying LCRs include the RNA- and DNA-binding proteins related to gene regulation [90]. LCRs seem to be significant even in prokaryotes since they have various functional roles and are highly conserved [91]. The basic consideration about LCRs is that they can undergo phase separation out of aqueous solutions to form either liquid-like droplets (LLDs) or amyloid-like reversible polymers upon in vitro incubation [92,93]. Especially in the case of RNA-binding protein hnRNPA1, the LCRs are sufficient to drive stress granule assembly, leading to pathological fibrillization [94]. Nevertheless, the process of phase separation in cellular processes as the biogenesis of membraneless cellular compartments should be viewed with skepticism [95]. Studies of isolated desmin and NFL head domains revealed that they are capable of shelf-interaction and they could form labile β-strand–enriched amyloid-like polymers under phase separation [52]. In *Drosophila*, the tail domain of the atypical tropomyosin Tm1-I/C, which presents several of the intermediate filament properties [96], forms labile and dynamic β-strand conformations (amyloid-like polymers) during the process of assembly [97]. LCRs are capable of forming secondary structures such as helixes and sheets [98], alongside the formation of toxic amyloids and aggregation in diseases [99], as it has been shown for desmin and heart failure [100,101,102] or keratin-8 and liver disease [103]. In addition, keratins are highly enriched in LARKS (low-complexity aromatic rich kinked segments), a structural motif which exhibits a kinked β-sheet structure where, upon polymerization, the aromatic residues interact in an intra- and inter-sheet manner [104], as has been proposed in the model of K1/K10 keratin assembly [55]. LARKS have been found in several proteins with LCRs [104].

Interestingly, atomic structures derived from microcrystallography studies of various amyloid fibrils reveal stacked, identical layers of protein monomers that form β-sheets. These β-sheet bilayers form a tightly interdigitating “steric zipper” [105]. The highly stable bonds which grow likely contribute to the resistance of amyloid fibrils to disaggregation and proteolytic degradation. In contrast to LARKS, amyloid fibrils are irreversible formations.

## 2. Discussion—Conclusions

Even though there is no crystallographic information specific to the head domain of intermediate filaments, the data so far indicate that maybe they are constituted by structural order, such as the pre-coil domain in several type III and IV IFs [1], or β-sheet formations in desmin [61]. The head domain of desmin has been predicted to consist of α helical motifs and β sheets [60]. On the other hand, the predictions about the octamer keratin K1/K10 assembly suggest the formation of an aromatic zipper mechanism that brings together two tetramers through the interaction of glycine loop regions of the head and tail domains [55]. Moreover, the fact that even in early stages of assembly, like in dimer formation, the head domains of vimentin or keratin fold back and interact with coil1A [27,41] emphasizes the dynamic nature of the head domain. Additionally, the known structural data from the low-complexity region of IF protein tail domains indicate the existence of a secondary structure. Specifically, the lamin tail domain consists of an Ig-like fold motif and, in dimer formation, those two motifs interact and create a continuous β-fold sheet structure, as is indicated in a crystallization study [57], while based on EPR analysis, the vimentin tail domain has been proposed to form a β-hairpin motif [59], derived from a conserved sequence motif among type III IFs [106]. During dimerization, two such motifs interact to shape a continuous β-sheet conformation [32,59]. Taken together, all of the above enhance the hypothesis of a certain structural order, flexibility, and dynamic framework of the terminal domains of IFs.

The head domain is necessary for the proper assembly and interactions of IFs [1] with other proteins or organelles. Phosphorylation, a common PTM, which regulate the dynamic assembly–disassembly process [72], could also regulate these interactions. Interestingly, some interactions seem to occur in a similar pattern, like in the case of desmin head domain interactions with NDUFS2 and saposin D, as indicated by an in silico analysis [60]. More specifically, the residues that contributed to both interactions through H-bonds are Arg16 and Ser32 [60]. R16 is included in the preserved motif “SSYRRTFGG” [31], and mutations in this motif could cause serious defects as in the case of desmin gene mutations S13F and R16C, which are linked to cardiomyopathy and atrioventricular block [107,108]. Desmin could also bind to desmoplakin and plectin by using a different binding region, referring to the C-terminal and N-terminal of the rod domain, respectively, and the same applies to vimentin [70,109]. This may provide insights into which intermediate filaments participate in multiple and non-competitive molecular interactions with various cytolinkers to strengthen their connections. It should be noted that interactions are stronger with full-length IFs. In addition, most of the IF proteins interact with nucleic acids through their head domains and, in this way, may affect nuclear chromatin [84]. Thus, for these fine-tuning and orchestrated processes, some kind of structural order is necessary, in contrast to the tendency of β-sheet conformations to form amyloid-like toxic aggregations [100,101,102,103], as it occurs in the LCRs of other kind of proteins [99]. This flexibility feature may explain the wide functionality of IF proteins in health and disease [76]. Therefore, a deeper understanding of the head and tail domains’ dynamic structure could provide insights into IF protein functions.

## Data Availability

Data are available upon request.
